# Clinically Relevant Mutations of Mycobacterial GatCAB Inform Regulation of Translational Fidelity

**DOI:** 10.1128/mBio.01100-21

**Published:** 2021-07-06

**Authors:** Yang-Yang Li, Rong-Jun Cai, Jia-Ying Yang, Tamara L. Hendrickson, Ye Xiang, Babak Javid

**Affiliations:** a Centre for Global Health and Infectious Diseases, Tsinghua University School of Medicine, Beijing, China; b Department of Chemistry, Wayne State University, Detroit, Michigan, USA; c Beijing Advanced Innovation Centre for Structural Biology, Beijing Frontier Research Centre for Biological Structure, Tsinghua University School of Medicine, Beijing, China; d Division of Experimental Medicine, University of California, San Francisco, California, USA; New York University School of Medicine

**Keywords:** GatCAB, translational fidelity, mycobacterium, transamidosome, mistranslation, *Mycobacterium tuberculosis*

## Abstract

Most bacteria employ a two-step indirect tRNA aminoacylation pathway for the synthesis of aminoacylated tRNA^Gln^ and tRNA^Asn^. The heterotrimeric enzyme GatCAB performs a critical amidotransferase reaction in the second step of this pathway. We have previously demonstrated in mycobacteria that this two-step pathway is error prone and translational errors contribute to adaptive phenotypes such as antibiotic tolerance. Furthermore, we identified clinical isolates of the globally important pathogen Mycobacterium tuberculosis with partial loss-of-function mutations in *gatA*, and demonstrated that these mutations result in high, specific rates of translational error and increased rifampin tolerance. However, the mechanisms by which these clinically derived mutations in *gatA* impact GatCAB function were unknown. Here, we describe biochemical and biophysical characterization of M. tuberculosis GatCAB, containing either wild-type *gatA* or one of two *gatA* mutants from clinical strains. We show that these mutations have minimal impact on enzymatic activity of GatCAB; however, they result in destabilization of the GatCAB complex as well as that of the ternary asparaginyl-transamidosome. Stabilizing complex formation with the solute trehalose increases specific translational fidelity of not only the mutant strains but also of wild-type mycobacteria. Therefore, our data suggest that alteration of GatCAB stability may be a mechanism for modulation of translational fidelity.

## INTRODUCTION

Protein synthesis is an essential but error-prone biological process (1–3). Recent evidence suggests that optimal translational fidelity is not necessarily as high as possible. This consequence is not just due to reasons of efficiency, i.e., a trade-off between speed and accuracy of protein synthesis, but also because translational errors, mistranslation, may allow adaptation to hostile environments, particularly in contexts of environmental stress (3–5). Nonetheless, excessive mistranslation is harmful. Therefore, mechanisms for “tuning” of optimal—and context-specific—translational fidelity are required. Multiple mechanisms for microbial adaptive mistranslation have been proposed (2, 3). Most bacterial species, including the majority of pathogens, use an indirect tRNA aminoacylation pathway due to their lack of specific glutaminyl or asparaginyl tRNA synthetases or both (6). In mycobacteria, which lack both glutaminyl and asparaginyl synthetases, we have previously shown that baseline error rates associated with the pathway (i.e., glutamine to glutamate and asparagine to aspartate) are ∼0.2 to 1%/codon, orders of magnitude higher than equivalent errors in Escherichia coli, which lacks the pathway (7, 8). Furthermore, elevated mistranslation in this pathway causes increased tolerance to the first-line antimycobacterial drug rifampin due to gain-of-function protein variants arising from mistranslation of critical residues in the drug target of rifampin, the beta subunit of RNA polymerase (7, 9). Consistently, reducing errors in this pathway with the small-molecule kasugamycin increases susceptibility to rifampin in Mycobacterium tuberculosis, both in axenic culture and in animal infection (10), confirming the critical role for mistranslation in mycobacterial rifampin tolerance.

In the first step of the two-step indirect pathway, a nondiscriminatory glutamyl- (ND-GluRS) or aspartyl-tRNA synthetase (ND-AspRS) mischarges tRNA^Gln^ to form Glu-tRNA^Gln^ and tRNA^Asn^ to form Asp-tRNA^Asn,^ respectively (11, 12). These mischarged tRNAs are specifically recognized by the heterotrimeric amidotransferase GatCAB, which converts them to Gln-tRNA^Gln^ and Asn-tRNA^Asn^, respectively. Mutations in *gatCAB* are surprisingly common and have been identified in a substantial minority of clinical isolates of M. tuberculosis*-*sequenced genomes; several of these mutations cause both elevated rates of mistranslation and rifampin antibiotic tolerance (9). This is all the more remarkable since these mutants were in disease-causing strains isolated from patient samples, suggesting that mycobacteria are surprisingly plastic and tolerant to elevated translational error arising from this pathway.

Bacterial GatCAB is a heterotrimeric, glutamine-dependent enzyme complex composed of GatA, GatB, and GatC. Based on primary sequence analysis, reported crystal structures, and experimental analyses of bacterial GatCAB (13–15), GatA serves as the glutaminase subunit for ammonia production (11), GatB is responsible for activation and transamidation of the misacylated tRNA (16), and GatC is a small scaffold protein that helps stabilize the complex (13). In some bacteria, GatCAB forms a ternary complex with ND-AspRS and possibly with ND-GluRS and tRNA^Asn^ or tRNA^Gln^, respectively, termed the transamidosome (17–22). It has been proposed that due to the physical proximity of the nondiscriminatory synthetase (the source of potential translational error) and GatCAB (which corrects mischarged tRNA complexes), the transamidosome may promote the efficiency of and minimize errors due to this pathway. Here, we biochemically and biophysically analyze mycobacterial GatCAB as well as two mutant enzymes. We show that the mutations primarily function to destabilize the enzyme and the ternary transamidosome complex. Stabilization of the complex with trehalose decreases mistranslation rates in both mutant and wild-type mycobacteria, suggesting that enzyme and transamidosome stability are mechanisms for regulating translational fidelity in mycobacteria.

## RESULTS

### Mutated *gatA* identified from clinical isolates does not dramatically alter GatCAB enzymatic function.

We wished to characterize the glutaminase and amidotransferase activities of mycobacterial GatCAB. We also wanted to compare wild-type enzyme function with two *gatA*-mutated enzymes identified in our prior study from clinical isolates of Mycobacterium tuberculosis, GatA-K61N and GatA-G444S (9). In that study, we showed that these mutations conferred substantially elevated rates of specific translational error in mycobacteria, which, in turn, resulted in enhanced antibiotic tolerance (9). However, the molecular mechanisms by which these mutations resulted in elevated mistranslation rates were not known. The GatA subunit of GatCAB serves as a glutaminase, liberating ammonia from glutamine, which is transferred to GatB via a hydrophilic tunnel, where it is used to amidate the misacylated Glu-tRNA^Gln^ or Asp-tRNA^Asn^ substrate (12, 13). The glutaminase reaction also results in generation of glutamate from glutamine. Using a commercial luminometric assay for glutamate, we measured the glutaminase activity (Fig. S1 in the supplemental material) of the wild-type and two mutant enzymes in the presence of Asp-tRNA^Asn^ (Table 1). Although there were some observable differences between the *K_m_* of the wild-type and, in particular, the GatCAB-K61N enzyme, the *k*_cat_/*K_m_* of the three enzymes were broadly similar (Table 1). Next, we measured the amidotransferase activity of the three enzymes using Asp-tRNA^Asn^ as a substrate using a modified ^32^P/P1 nuclease assay followed by thin-layer chromatography (23, 24) and Fig. S2. Again, the enzymatic activity of all three enzymes was similar, with the two mutant enzymes actually showing slight increases (∼25%) in *k*_cat_/*K_m_* compared with the wild-type enzyme (Table 2). Together, these data suggest that the elevated mistranslation rates observed in mycobacteria harboring these clinically derived mutations cannot be explained by decreased enzymatic function due to these mutations.

**TABLE 1:** Kinetic data for WT, G444S, and K61N M. tuberculosis GatCAB glutaminase activity^*a*^

Enzyme	*K_m_*, Gln (mM)	*k*_cat_ (s^−1^)	*k*_cat_/*K_m_*, Gln (s^−1^/mM)
WT	0.13 ± 0.02	0.95 ± 0.04	7.31
G444S	0.18 ± 0.03	0.90 ± 0.04	5.00
K61N	0.24 ± 0.04	1.53 ± 0.08	6.38

a Reactions were carried with 5 μM Asp-tRNA^Asn^ containing 20 nM GatCAB enzyme. Values represent means ± SD of independent biological replicates, the number of which are listed in Fig. S1D.

**TABLE 2:** Kinetic data for WT, G444S, and K61N M. tuberculosis GatCAB amidotransferase activity^*a*^

Enzyme	*K_m_*, asp-tRNA^Asn^ (μM)	*k*_cat_ (s^−1^)	*k*_cat_/*K_m_* (asp-tRNA^Asn^) (s^−1^/μM)
WT	0.30 ± 0.10	0.063 ± 0.004	0.21
G444S	0.16 ± 0.05	0.044 ± 0.002	0.28
K61N	0.31 ± 0.13	0.085 ± 0.008	0.27

a Comparison of *K_m_* (with Asp-tRNA^Asn^ as the substrate), *k*_cat_, and *k*_cat_/*K_m_* between the GatCAB variants WT (*n *= 4), G444S (*n *= 4), and K61N (*n *= 3). We added 1 mM Gln and 4 mM ATP with 10 nM enzyme. Values represent means ± SD of independent biological replicates as shown above.

10.1128/mBio.01100-21.3 FIG S1 Glutaminase assay of WT, G444S, and K61N M. tuberculosis GatCAB. Michaelis-Menten curves of WT (A), G444S (B), and K61N (C) GatCAB glutaminase activity. Reactions were carried with 5 μM Asp-tRNA^Asn^, containing ∼20 nM GatCAB. GraphPad Prism was used to calculate kinetic parameters by nonlinear regression. (D) Error bars represent the standard deviation of independent biological replicates, the number of which are listed. Download FIG S1, PDF file, 0.1 MB. Copyright © 2021 Li et al. 2021 Li et al. https://creativecommons.org/licenses/by/4.0/ This content is distributed under the terms of the Creative Commons Attribution 4.0 International license.

10.1128/mBio.01100-21.4 FIG S2 GatCAB amidotransferase assay with ^32^P-labeled tRNA. (A) Representative phosphorimage of the separation of Asn-[α-^32^P]AMP, Asp-[α-^32^P]AMP, and [α-^32^P]AMP, by PEI cellulose chromatography. Aliquots (5 μl) of the amidotransferase reaction at 37°C (10 nM GatCAB from M. tuberculosis, 0.5 μM Asp-tRNA^Asn^, 1 mM Gln, 4 mM ATP, 25 mM KCl, 8 mM MgCl_2_, and 40 mM HEPES-KOH pH 7.5) were taken at the time points indicated and quenched/digested at 37°C with 5 μl of 100 mM sodium citrate, pH 4.7, and 0.66 mg/ml of nuclease P1. Digested samples (3 μl) were then spotted onto a 20- by 20-cm PEI-cellulose glass plate, which was developed in 10 mM ammonium chloride and 5% acetic acid for ∼2 h. Aliquot from no-GatCAB reaction at 120 seconds was taken as the background control. Michaelis-Menten curves of WT (*n *= 4) (B), G444S (*n *= 4) (C), and K61N (*n *= 3) (D) GatCAB are shown for their amidotransferase activity. GraphPad Prism was used to calculate kinetic parameters by nonlinear regression. Error bars represent the standard deviation of independent biological replicates. Download FIG S2, PDF file, 0.3 MB. Copyright © 2021 Li et al. 2021 Li et al. https://creativecommons.org/licenses/by/4.0/ This content is distributed under the terms of the Creative Commons Attribution 4.0 International license.

### GatCAB stability is compromised by clinical mutations.

Having demonstrated that loss of enzymatic activity does not explain the partial loss of function observed in bacteria with these two mutations, we proceeded to test another aspect of GatCAB function, that of heterotrimer stability. In our earlier study, we had performed an *in vitro* forward genetic selection and screen for high-mistranslation mutants and identified several mutations in *gatA* (9). Although the mutations were in *gatA*, (wild-type) GatB protein stability was also lower in a pulse-chase experiment, indirectly implicating heterotrimer stability in those *in vitro*-selected mutants (9). We tested two aspects of enzyme stability using thermal ramp assays, conformational stability, determined by measuring complex unfolding through intrinsic fluorescence, denoted Tm1 (Fig. S3A), and colloidal stability by detecting particle aggregation via static light scattering, denoted Tagg_266_ (Fig. S3B). Both mutant enzymes showed lower colloidal stability, with stability of wild type (WT) greater than GatCAB-K61N, which is greater than GatCAB-G444S. With regard to conformational stability, only the GatCAB-G444S was significantly less stable than the other two enzymes (Table 3).

**TABLE 3:** Thermostability of GatCAB variants (Tm1 and Tagg_266_)^*a*^

Enzyme	Thermostability (°C) of:			
	Tm1		Tagg_266_	
	Mean ± SD	*P* value	Mean ± SD	*P* value
WT	47.2 ± 0.25	NA	46.8 ± 0.28	NA
G444S	40.9 ± 0.25	<0.001	44.1 ± 0.02	<0.001
K61N	48.0 ± 0.44	0.06	44.7 ± 0.17	<0.001

a Comparison of Tm1 and Tagg_266_ between the GatCAB variants WT (*n *= 3), G444S (*n *= 3), and K61N (*n *= 3). Samples were tested at 1.7 mg/ml. *P* values show comparison by Student's *t* test to WT GatCAB. NA, not applicable.

10.1128/mBio.01100-21.5 FIG S3 Thermostability assay of M. tuberculosis GatCAB. The assay was conducted on an UNcle instrument (Unchained Labs) by monitoring thermal melting and aggregation over a temperature range. (A) Representative Tm1 characterization of WT GatCAB using intrinsic fluorescence. At each temperature, the barycentric mean (BCM) of the intrinsic fluorescence spectra was measured and plotted against temperature to generate the BCM curve (blue). First-order derivative of the BCM curve was calculated and plotted against temperature (green). The temperature corresponding to the first peak of the first-order derivative curve was defined as Tm1, as shown by the dotted line (green). (B) Representative Tagg_266_ characterization of WT GatCAB using static light scattering. At each temperature, the intensity of static light scattering at 266 nm (as represented by SLS 266 nm) was measured and plotted against temperature to generate an aggregation curve. Aggregation onset temperature (Tagg_266_) of each protein was calculated by UNcle Analysis software, as shown by the dotted line. Download FIG S3, PDF file, 0.1 MB. Copyright © 2021 Li et al. 2021 Li et al. https://creativecommons.org/licenses/by/4.0/ This content is distributed under the terms of the Creative Commons Attribution 4.0 International license.

### Mycobacterial ND-AspRS and GatCAB form a stable asparagine-transamidosome in a tRNA^Asn^-dependent manner.

**FIG 1:**
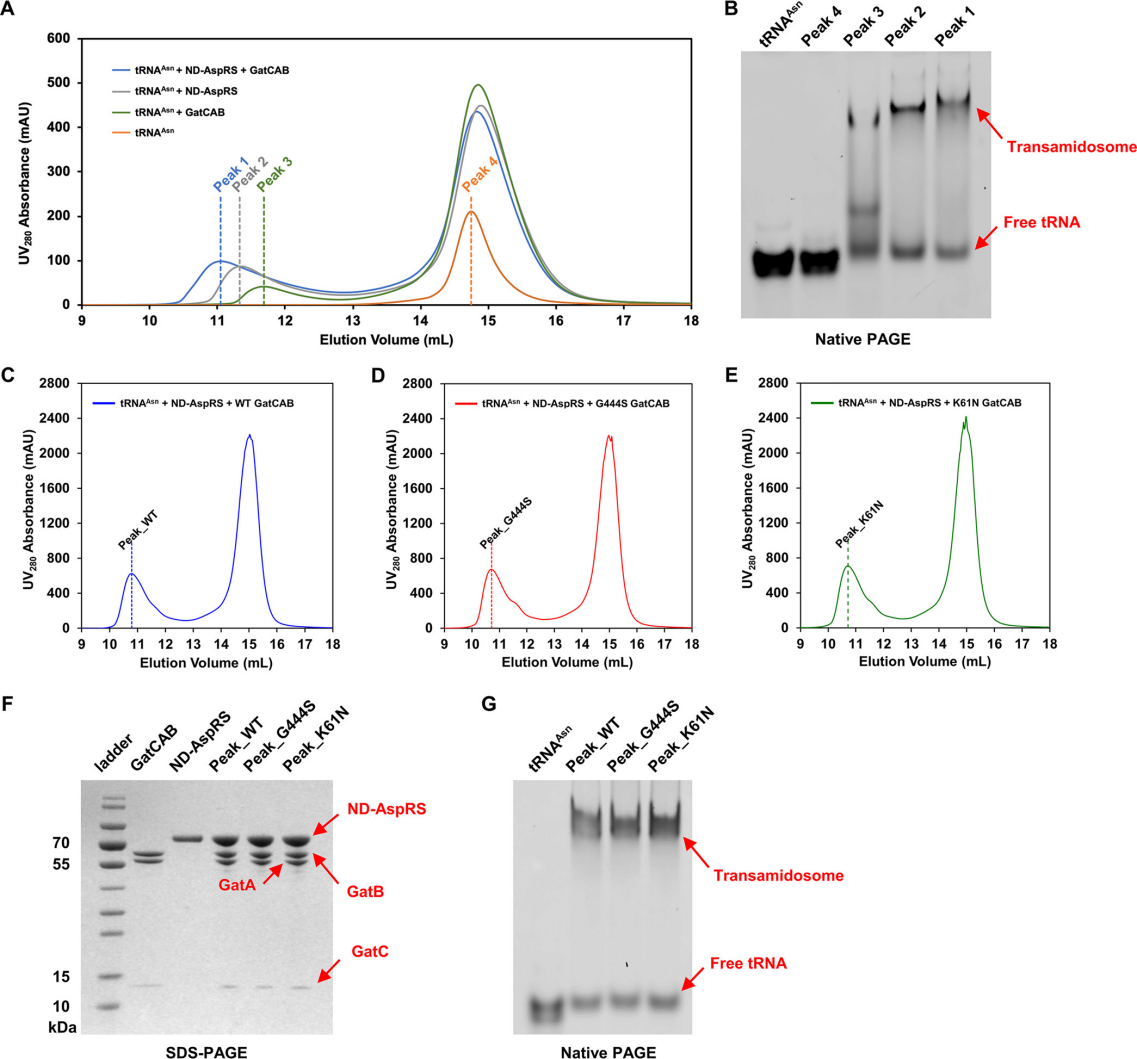
Formation, verification, and characterization of the M. tuberculosis Asn-transamidosome. (A) Size exclusion chromatography of tRNA^Asn^, ND-AspRS, and WT GatCAB, confirming formation of the Asn-transamidosome as shown by earlier elution peak. (B) Native PAGE analysis of peaks 1 to 4 from panel A, stained with SYBR green II. (C to E) Size-exclusion profiles of Asn-transamidosomes with WT GatCAB (C), GatCAB-G444S (D), and GatCAB-K61N (E), as well as tRNA^Asn^ and ND-AspRS. (F) SDS-PAGE analysis of the peaks illustrated in panels C to E alongside WT GatCAB and ND-AspRS. (G) Native PAGE of the peaks illustrated in panels C to E alongside tRNA^Asn^ and stained with SYBR green II.

The transamidosome is a ternary complex of GatCAB, nondiscriminatory synthetase, and tRNA. Prior studies suggest that several organisms form relatively stable asparaginyl-transamidosomes (17–20, 24); however, such a complex has never been characterized for a mycobacterial system. We used size exclusion chromatography to determine whether M. tuberculosis also formed a stable asparaginyl transamidosome (Asn-transamidosome). ND-AspRS, tRNA^Asn^, and GatCAB were coeluted from a size exclusion column isolated with a shorter elution time than any of the individual components (Fig. 1A and C). Native PAGE analysis of the individual peaks confirmed a molecular mass shift of the tRNA-containing fraction, in keeping with Asn-transamidosome assembly (Fig. 1B). All three components were necessary for transamidosome formation since there did not appear to be complex formation between GatCAB and ND-AspRS alone (Fig. S4). These two enzymes eluted with similar retention times as each other, likely reflecting similar masses of GatCAB (117 kDa) and the natural dimeric state (25) of ND-AspRS (130 kDa) and with no shift observed when combined. Both GatCAB-K61N and GatCAB-G444S were also able to form Asn-transamidosomes (Fig. 1D to G).

10.1128/mBio.01100-21.6 FIG S4 Formation of the M. tuberculosis Asn-transamidosome is tRNA dependent. Gel filtration conducted with free ND-AspRS, free GatCAB, a mixture of ND-AspRS and GatCAB, and a mixture of ND-AspRS, GatCAB, and tRNA^Asn^. We used 4 μM ND-AspRS, 2 μM WT GatCAB, and 2 μM tRNA^Asn^. Download FIG S4, PDF file, 0.1 MB. Copyright © 2021 Li et al. 2021 Li et al. https://creativecommons.org/licenses/by/4.0/ This content is distributed under the terms of the Creative Commons Attribution 4.0 International license.

### The GatCAB-G444S-transamidosome is relatively unstable.

**FIG 2:**
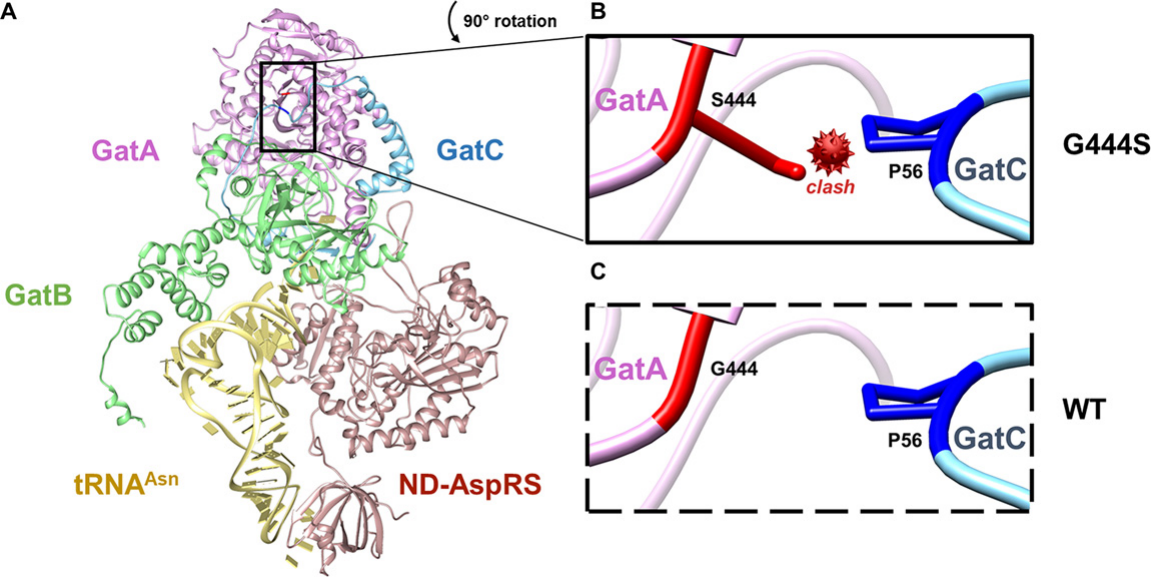
Structural modeling of the M. tuberculosis Asn-transamidosome explains complex instability due to GatA-G444S. (A) Predicted structural model of the Asn-transamidosome. M. tuberculosis GatA, GatB, GatC, and ND-AspRS are colored magenta, green, cyan, and brown, respectively. tRNA^Asn^ (yellow) docked onto the structure is from the P. aeruginosa asparagine-transamidosome (PDB ID 4WJ3). The site of the 444 residue of GatA is shown in the boxes. (B) GatA-G444S; (C) GatA-WT. G444 and S444 are colored red, while P56 from GatC is blue. The box shows a magnified 90° counterclockwise rotated view of the mutation site.

To better understand the impact of the GatCAB-K61N and GatCAB-G444S mutations on transamidosome function, we modeled the structure of the mycobacterial Asn-transamidosome based on the crystal structure of the Pseudomonas aeruginosa Asn-transamidosome (20). Compared with the wild-type enzyme, the substitution of a serine for the glycine at position 444 in mycobacterial GatA resulted in a predicted steric clash with a proline in the GatC subunit (Fig. 2). No such steric hindrance was observed for GatCAB-K61N (61K/N is located at the GatA surface; data not shown). In keeping with these models, when we measured the stability of the wild-type and mutant enzyme Asn-transamidosomes by thermal ramp assay, colloidal stability was significantly compromised for GatCAB-G444S but not GatCAB-K61N (Table 4).

**TABLE 4:** Thermostability of Asn-transamidosome variants (Tm1 and Tagg_266_)^*a*^

Asn-transamidosome complex enzyme	Thermostability (°C) of:			
	Tm1		Tagg_266_	
	Mean ± SD	*P* value	Mean ± SD	*P* value
WT	42.0 ± 0.56	NA	43.6 ± 0.44	NA
G444S	41.2 ± 1.17	0.16	43.0 ± 0.44	0.03
K61N	41.7 ± 0.97	0.51	43.7 ± 0.21	0.44

a Comparison of Tm1 and Tagg_266_ between the transamidosome variants WT (*n *= 7), G444S (*n *= 6), and K61N (*n *= 7). Samples were tested at 0.5 mg/ml. *P* values show comparison by Student's *t* test to WT transamidosome.

### Stabilizing GatCAB and the Asn-transamidosome with trehalose increases specific translational fidelity.

**FIG 3:**
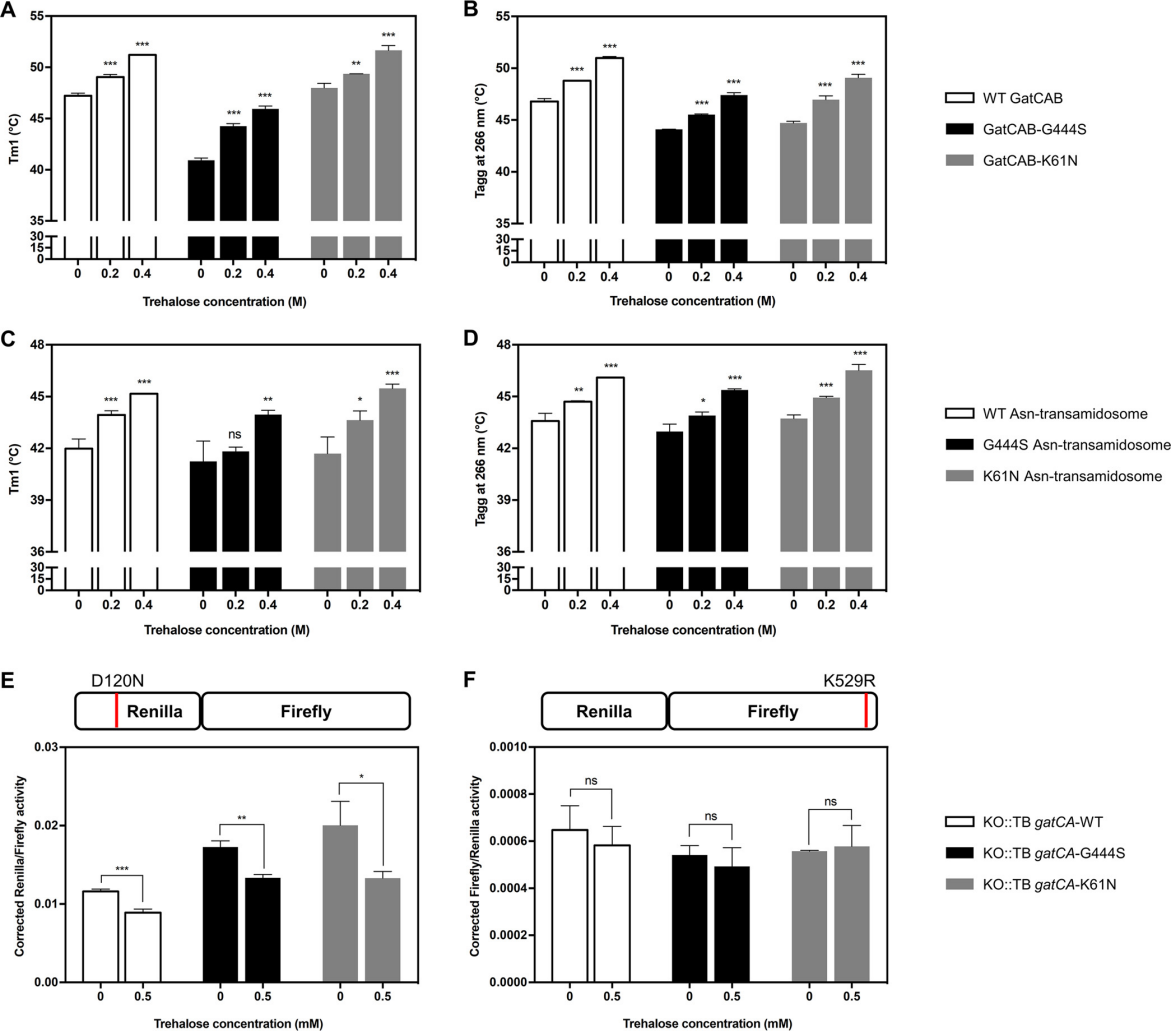
Stabilizing GatCAB and the Asn-transamidosome increases specific mycobacterial translational fidelity. *In vitro* thermostability assay of WT and mutant GatCAB of Tm1 (A) and Tagg_266_ (B) and Asn-transamidosomes of Tm1 (C) and Tagg_266_ (D) with and without trehalose at indicated concentrations; *n* ≥ 3 biological replicates. (E and F) Mistranslation rates, expressed as corrected *Renilla*/firefly (E) and corrected firefly/*Renilla* (F) of M. smegmatis expressing M. tuberculosis wild-type or mutated *gatCA* in cultures containing trehalose at indicated concentrations (see Materials and Methods). The cartoon above each bar chart depicts the type of error measured preribosomal error due to the indirect pathway (E) and ribosomal decoding errors (F). Error bars represent the standard deviation of three independent cultures. Asterisks in each case represent level of significance as determined by Student's *t* test compared with the no-trehalose group. ns, *P* > 0.05; *, *P* < 0.05; **, *P* < 0.01; ***, *P* < 0.001.

Our biochemical and biophysical data suggested that *gatA* mutations that impact translational fidelity act via destabilization of the GatCAB heterotrimer or Asn-transamidosome or both. To further explore this model, we decided to attempt to stabilize the complexes with the sugar trehalose, which has been previously demonstrated to have a buffering effect on potentially deleterious or destabilizing mutations (26, 27). We repeated the thermostability assays in the absence or presence of trehalose. Trehalose significantly increased the stability of both GatCAB-K61N and GatCAB-G444S heterotrimers (Fig. 3A and B) as well as the corresponding Asn-transamidosomes (Fig. 3C and D). Intriguingly, trehalose also increased the stability of the wild-type GatCAB heterotrimer and Asn-transamidosome (Fig. 3A to D).

To translate these findings to an *in vivo* setting, we decided to test the impact of GatCAB and transamidosome stabilization by trehalose on translational fidelity in mycobacteria. For pragmatic reasons, including the lack of a luminometer in the biosafety level 3 facility, we tested mistranslation rates in the nonpathogenic Mycobacterium smegmatis, in which the native *gatCA* locus had been deleted and replaced elsewhere on the chromosome with wild-type or mutant *gatCA* from M. tuberculosis (9). Using a set of dual-luciferase reporters (7, 28), we decided to measure two different forms of translational error representing two distinct pathways. To quantify errors generated by the preribosomal indirect tRNA aminoacylation pathway, we measured mistranslation of aspartate for asparagine using a reporter in which a critical aspartate residue in *Renilla* luciferase was mutated to asparagine (29). We also measured ribosomal decoding errors using the same *Renilla*/firefly luciferase system, but with a mutation of a critical lysine in firefly luciferase (28, 30, 31). We reasoned that if trehalose specifically stabilized GatCAB and the Asn-transamidosome, only the preribosomal error rate should be lowered. Indeed, asparagine-to-aspartate mistranslation rates were significantly lower with trehalose, not only in the strains expressing mutant *gatA* but also in the strain expressing wild-type M. tuberculosis *gatA* (Fig. 3E). In contrast, there was no significant impact of trehalose supplementation on rates of ribosomal decoding errors in any of the strains, suggesting that trehalose was not influencing translational fidelity or reporter function in a general manner (Fig. 3F). These data suggest that stabilization of wild-type, as well as mutant GatCAB and/or the Asn-transamidosome, increases translational fidelity of the indirect tRNA aminoacylation pathway and suggests this may be a physiologically relevant mechanism by which mistranslation rates are tuned in response to environmental triggers and stressors (7, 9).

## DISCUSSION

In this study, we chose to focus on two mutations in *gatA* identified from tuberculosis patient samples and previously confirmed to cause increased rates of specific mistranslation and antibiotic tolerance (9). Both the GatCAB-K61N and GatCAB-G444S mutations destabilized the heterotrimeric enzyme as measured by thermal ramp assays, but only the GatCAB-G444S-Asn-transamidosome was less stable than wild type using the same assays, and the relative difference in stability of the transamidosome was smaller than for just GatCAB. There are three possible explanations for these findings. Prior to assay by thermal ramp, the Asn-transamidosomes were first purified by gel filtration. This would preselect the samples for stable complexes since highly unstable complexes would not elute as the higher-molecular-weight peak. Additionally, the assembly of the ternary complex itself may further stabilize (17) and mitigate against the destabilizing mutation. By bringing together all three components required for the generation of cognately aminoacylated tRNA^Asn^, the Asn-transamidosome has been proposed to increase fidelity of translation in the indirect pathway (17). The greater instability of GatA-G444S may, nonetheless, have resulted in unstable complex in the downstream assays. Given that GatA-G444S was more unstable than GatA-K61N in our biophysical studies, we might expect the mycobacterial strain expressing the G444S mutation to have a higher mistranslation rate than one expressing K61N, but this was not observed (Fig. 3E). This may be due to the heterologous expression of Mtb-GatCA in M. smegmatis masking observed differences or the inability of our structural modeling and biophysical studies to capture more dynamic perturbations caused by the GatA-K61N mutation. More generally, our studies confirm not only that mutations that destabilize the ternary complex also cause increased mistranslation, but that stabilizing the complex, in this case with the disaccharide trehalose, improves translational fidelity, demonstrating the validity of the model in living bacteria for the first time.

Our kinetic studies found that the enzymatic function of the GatCAB-K61N and GatCAB-G444S mutants was broadly similar to the wild-type enzyme, although there were subtle decreases in glutaminase activity. However, glutaminase is not the rate-limiting step in GatCAB function, and the mutant enzymes actually had slightly increased amidotransferase activity. One recent study performed a modified glutaminase assay for mycobacterial GatCAB (32). However, they did not conduct kinetic analyses, and their assay conditions—extremely high enzyme concentrations and incubation over several hours prior to measurements—make comparison with our studies difficult. More generally, our kinetic studies suggest that mycobacterial GatCAB is less efficient than enzymes previously characterized from Neisseria meningitidis and Moraxella catarrhalis, although more similar to that of Helicobacter pylori (19, 23, 33, 34). Relatively inefficient amidotransferase activity by mycobacterial GatCAB may explain the observed high basal mistranslation associated with this pathway in mycobacteria; however, it should be noted that the equivalent error rates in the other organisms have not been measured. It may also explain why individual bacilli within an isogenic population of wild-type mycobacteria that have lower expression of (wild-type) *gatCAB* have higher mistranslation rates, suggesting that GatCAB expression is limiting in mycobacteria (9). The activity of H. pylori GatCAB was stimulated by the presence of an additional protein, Hp0100 (QueH), which also allowed the formation of a stable, Hp0100-dependent but tRNA-independent Asn-transamidosome (24). It is possible that a functionally equivalent protein exists in mycobacteria, boosting both GatCAB activity and transamidosome stability. However, it should be noted that unlike *Helicobacter* (19, 24), the mycobacterial Asn-transamidosome is stable with just the tRNA, synthetase, and GatCAB components.

Small molecules targeting enzymatic inhibition of GatCAB have been discovered (35, 36), but none have been validated against bacteria. Our work suggests that compounds that inhibit GatCAB could act not just by inhibiting enzyme synthesis but also by destabilizing GatCAB or Asn-transamidosome complex formation. Alternatively, compounds that hyperstabilize the complex may decrease mistranslation and consequently reduce “adaptive mistranslation,” analogous to the effect of kasugamycin on rifampin susceptibility (10). Given the presence of the indirect tRNA aminoacylation pathway in the majority of human pathogens, further studies in targeting this pathway may be warranted.

Studies of naturally occurring mutations in the genes encoding GatCAB can inform complex function. Mutations in all three components of human mitochondrial GatCAB have been associated with neonatal lethal cardiomyopathy (37). This may suggest that mammalian mitochondria are less tolerant of partial loss-of-function mutations in GatCAB, but it is noteworthy that there was a clear gradient in toxicity caused by the mutations in tissues, with cardiac muscle severely affected and almost no phenotype in patient fibroblasts (37). In contrast, bacteria seem to better tolerate mutations in *gatCAB*. Penicillin-resistant isolates of M. catarrhalis have an insertion of a beta-lactamase gene between *gatA* and *gatB* in the *gatCAB* operon. The insertion alters the C terminus of GatA, resulting in a subtle change in enzyme activity, but with no apparent loss in bacterial viability (33). Analysis of the two mycobacterial *gatA* mutations presented here suggest a potential mechanism by which mycobacteria may tune translational fidelity in response to environmental cues (7). Having identified that the mutations destabilize GatCAB and the Asn-transamidosome, we sought to stabilize the complexes with trehalose. Trehalose is a naturally occurring glucose disaccharide that is found in bacteria, plants, and some invertebrates but absent in mammals. Trehalose is essential for mycobacterial growth (38) and cell wall component synthesis (39, 40) and abundant in mycobacteria, making up 1 to 3% of dry weight. Trehalose has also been implicated in stress response and buffering of deleterious mutations, acting as a chemical chaperone (26, 27, 41). We found that trehalose not only stabilized mutant GatCAB but also wild-type GatCAB and the Asn-transamidosome. Importantly, trehalose supplementation of cultures of mycobacterial strains expressing wild-type GatCAB caused a reduction in translational errors specifically for the indirect tRNA aminoacylation pathway. These data suggest that the wild-type GatCAB complex, Asn-transamidosome, or both may be inherently unstable and that stabilizing the complex, either via mass action by altering expression or via other mechanisms such as partner protein(s), may be a mechanism for tuning fidelity. Our work suggests that studying naturally occurring variations in essential members of the translation apparatus can inform mechanisms of protein synthesis regulation.

## MATERIALS AND METHODS

### Bacterial strains and growth conditions.

Details of constructed strains and primers used in this study are listed in Tables S1 and S2 in the supplemental material. E. coli strains were cultured in LB medium (BD Difco; catalog no. DF0402-07-0) or 2× YT medium with antibiotics as appropriate. All Mycobacterium smegmatis strains were cultured in Middlebrook 7H9 medium (BD Difco; catalog no. DF0713-17-9) supplemented with 0.2% glycerol, 0.05% Tween 80, and 10% ADS (albumin, dextrose, and salt) in the presence of hygromycin (50 μg/ml) or trehalose, as indicated. If not otherwise noted, cells were grown and maintained at 37°C with shaking.

10.1128/mBio.01100-21.1 TABLE S1 Bacterial strains used in this study. Download Table S1, DOCX file, 0.02 MB. Copyright © 2021 Li et al. 2021 Li et al. https://creativecommons.org/licenses/by/4.0/ This content is distributed under the terms of the Creative Commons Attribution 4.0 International license.

10.1128/mBio.01100-21.2 TABLE S2 Primers used in this study. Download Table S2, DOCX file, 0.02 MB. Copyright © 2021 Li et al. 2021 Li et al. https://creativecommons.org/licenses/by/4.0/ This content is distributed under the terms of the Creative Commons Attribution 4.0 International license.

### Cloning, expression, and purification of M. tuberculosis ND-AspRS.

The M. tuberculosis *aspS* gene (encoding ND-AspRS, start codon changed to ATG) containing 6×His tag fused at the C terminus was amplified by PCR with the appropriate primers (Table S2). The PCR product was digested with XbaI and HindIII and inserted into the corresponding sites of pET-28a(+) (Novagen; catalog no. 69864-3CN). The expression vector was transformed into E. coli Transetta(DE3) chemically competent cells (Transgen Biotech; catalog no. CD801). Cells were grown at 37°C in LB medium containing 50 μg/ml kanamycin and 34 μg/ml chloramphenicol, and protein expression was induced by adding IPTG (isopropyl-β-d-thiogalactopyranoside) to a final concentration of 1 mM at an optical density at 600 nm (OD_600_) of 0.4 to 0.6 for 4 h. Cells were harvested, resuspended in buffer A (50 mM NaH_2_PO_4_, pH 7.4, and 300 mM NaCl) containing 10 mM imidazole, 0.1 U/ml DNase I (Thermo Scientific; catalog no. EN0521), and InStab protease cocktail, EDTA-free (Yeasen Biotech; catalog no. 20123ES50). After sonication and centrifugation at 11,000 rpm (15,557 × *g*) for 75 min at 4°C, the supernatant was loaded onto the column with preequilibrated Ni-nitrilotriacetic acid (Ni-NTA) resin (Qiagen; catalog no. 30230). All the following purification processes were carried out at room temperature, with buffers containing protease inhibitor stored on ice. The column was washed with 10 mM and 40 mM imidazole in buffer A sequentially, and the protein was eluted in a stepwise manner with 80 mM, 150 mM, and 250 mM imidazole in buffer A, respectively. Only the 250-mM elution fractions were collected and exchanged into buffer B (50 mM NaH_2_PO_4_, pH 7.4, and 300 mM KCl) and concentrated using Amicon Ultra-15 30-kDa centrifugal filter device (Merck Millipore; catalog no. UFC903024) at 4°C. The final protein concentration was determined by Bradford reagent (Bio-Rad; catalog no. 5000205) and the purity checked by SDS-PAGE. For the aminoacylation assay, the protein was stored at −20°C in buffer B containing 50% glycerol. Otherwise, ND-AspRS was flash frozen in liquid nitrogen and stored at −80°C.

### Cloning, expression, and purification of M. tuberculosis GatCAB.

The M. tuberculosis *gatCA* gene (start codon of *gatC* and *gatA* changed to ATG) fused to an N-terminal Strep-tag II (amino acid sequence WSHPQFEK) and *gatB* gene containing a C-terminal 6×His tag were amplified by PCR with the appropriate primers (Table S2) and ligated to NcoI/PacI digested pETDuet-1 vector (Novagen; catalog no. 71146-3CN), using Gibson Assembly (New England Biolabs; catalog no. E2611). The expression vector was transformed into E. coli Transetta(DE3) chemically competent cells. Cells were grown at 37°C in LB medium containing 100 μg/ml ampicillin and 34 μg/ml chloramphenicol. Protein expression was induced by adding IPTG to a final concentration of 0.2 mM at an OD_600_ of 0.6 to 0.7, and bacteria were cultured for 16 h at 16°C. Buffers for all the following steps were added with protease inhibitor.

Cells were harvested, resuspended, sonicated, and centrifuged in the same manner as ND-AspRS purification. The protein complex was first purified by Ni-NTA affinity chromatography carried out at room temperature with buffers stored on ice. Clarified supernatant was bound to the column preequilibrated with buffer A containing 10 mM imidazole. After subsequent washing with 10 mM and 40 mM imidazole in buffer A, the proteins were eluted with buffer A containing 250 mM imidazole. The elution then went through buffer exchange for further purification, carried out at 4°C.

For the GatA glutaminase assay, the Ni-NTA eluate was exchanged into buffer C (50 mM Tris-HCl, pH 8.0, and 300 mM NaCl), concentrated, and applied to Strep-Tactin affinity chromatography column (IBA Lifesciences; catalog no. 2-1201-025) according to the manufacturer’s protocol. The elution buffer was buffer C containing 5 mM d-desthiobiotin (Sigma-Aldrich; catalog no. D1411). The protein was further purified by size exclusion chromatography using ÄKTA purifier (Cytiva) and an HK 16/40 column (Huiyan Bio; catalog no. HT16-40) packed with Sephacryl S-300 HR resin (Cytiva; manufacturer no. 17-0599-10), equilibrated with buffer C. Peak fractions eluted at the expected volume were pooled and concentrated. For other studies, the Ni-NTA eluate was exchanged into buffer D (20 mM HEPES-NaOH, pH 8.0, and 300 mM NaCl), concentrated, and applied to Strep-Tactin affinity chromatography according to the manufacturer’s protocol (IBA Life Sciences). The protein was eluted with buffer D containing 5 mM d-desthiobiotin, exchanged into buffer D, and concentrated.

The final protein concentration was determined by Bradford reagent or Qubit protein assay kit (Invitrogen; catalog no. Q33212), and the presence of all three subunits was verified by SDS-PAGE. For the GatCAB amidotransferase assay, the protein was stored at −20°C in buffer D containing 50% glycerol. For other studies, GatCAB was flash frozen in liquid nitrogen and stored at −80°C. GatCAB variants were generated using site-directed mutagenesis using appropriate primer pairs and purified to homogeneity as above.

### Preparation of M. tuberculosis tRNA^Asn^.

The T7 promoter and the Mtb tRNA^Asn^ gene were amplified using PCR with the appropriate primers (Table S2). The PCR product was digested with EcoRI and BamHI and inserted into the corresponding sites of pTrc99a (Tiandz; catalog no. 60908-6580). The expression vector was transformed into E. coli BL21(DE3) chemically competent cells (CWBio; catalog no. CW0809). Cells were grown at 37°C in 2× YT medium containing 100 μg/ml ampicillin to an OD_600_ of 0.8 before induction. Overexpression was induced by adding IPTG to a final concentration of 0.4 mM, and bacteria were cultured for 16 h at 37°C. Total nucleic acids were extracted with minor modifications of a previously described procedure (42). Cells were harvested and resuspended in 50 mM Tris-acetate, pH 7.8, 4 M guanidine thiocyanate, 15 mM β-mercaptoethanol, and 2% Triton X-100. After 15 min of incubation on ice, the mixture was mixed with an equal volume of 3 M sodium acetate, pH 6.5, incubated for another 15 min on ice, and centrifuged at 11,000 rpm (15,557 × *g*) for 30 min at 4°C. The lysate was precipitated with isopropanol followed by centrifugation at 11,000 rpm (15,557 × *g*) for 10 min at 4°C. The pellet was resuspended in 10 mM Tris-acetate, pH 7.8, and 1 mM EDTA, and the suspension was extracted twice with equal volumes of RNA extraction reagent (phenol/choloform/isoamyl alcohol, 25:24:1, pH < 5.0; Solarbio; catalog no. P1011). The aqueous layer was isopropanol precipitated. The pellet was redissolved in diethyl pyrocarbonate (DEPC)-treated water and incubated with 3 times volume of 100 mM Tris-HCl, pH 9.0, for 60 min at 37°C to ensure complete deacylation of the tRNA.

The mixture was isopropanol precipitated for subsequent tRNA enrichment based on the procedure of Spears et al. (43) Briefly, the total nucleic acid pool was mixed with 1 M MOPS (morpholinepropanesulfonic acid), pH 7.0, to a final concentration of 0.1 M and loaded onto a Qiagen-tip 2500 column (Qiagen; catalog no. 10083) preequilibrated with 2× 50 ml of fresh buffer E (50 mM MOPS, pH 7.0, 15% isopropanol, and 1% Triton X-100). After washing the column with 4× 50 ml of buffer F (50 mM MOPS, pH 7.0) containing 200 mM NaCl, the RNA was eluted with multiple fractions of 10 ml buffer F containing 650 mM NaCl. Ideal fractions of eluate (based on denaturing urea-PAGE; data not shown) were collected and precipitated with isopropanol, and pellets were stored at −20°C. This approach yielded a highly enriched stock of Mtb tRNA^Asn^ also containing E. coli tRNAs and other nucleic acids.

### Folding, quantification, and aminoacylation of tRNA^Asn^.

The experiment was performed as previously described (42) with some modifications. Prior to use for aminoacylation with aspartate, tRNA^Asn^ was dissolved in DEPC-treated water to the desired concentration and incubated in a 75°C water bath for 5 min. MgCl_2_ was added to a final concentration of 2 mM once the tRNA^Asn^ was allowed to cool slowly to 65°C. After the sample was cooled to below 45°C, it could be used for aminoacylation or stored at −20°C for later experiments.

The fraction of chargeable tRNA^Asn^ was confirmed for every batch prepared. Briefly, an aminoacylation assay with ND-AspRS was performed as follows: 100 μl aminoacylation assay with 20 mM HEPES-OH, pH 7.5, 2 mM ATP, 4 mM MgCl_2_, 100 μM aspartate, and 25 μCi/ml l-[2,3-^3^H] aspartate (PerkinElmer; catalog no. NET390V001MC). The reaction was initiated by adding 1 μM ND-AspRS followed by incubation at 37°C for 1 h. The assay was quenched by adding phenol-chloroform (pH < 5.0). The aqueous layer was isopropanol precipitated at −20°C for 2 h and then pelleted. The pellet was dissolved in 100 μl double-distilled water (ddH_2_O), from which aliquots of 10 μl were counted in vials with 3 ml OptiPhase HiSafe 3 scintillation fluid (PerkinElmer; catalog no. 1200-437). The fraction of charged tRNA^Asn^ was calculated based on the charged tritium-labeled aspartate incorporated into the pellet. Unlabeled Asp-tRNA^Asn^ was prepared as described above but without any tritium-labeled aspartate. The pelleted Asp-tRNA^Asn^ was dissolved in DEPC-treated water to 200 μM and stored in small aliquots at −20°C if not used immediately.

### GatA glutaminase assay.

The GatA glutaminase assay was conducted at 37°C in 40 mM HEPES-KOH, pH 7.5, 25 mM KCl, 8 mM MgCl_2_, and 5 μM Asp-tRNA^Asn^. The concentration of GatCAB was ∼20 nM. The reaction mixture for each assay was incubated on ice and then preequilibrated at 37°C for 2 min. Glutamine (final concentration varying from 50 to 2,000 μM) was added to initiate the reaction. At each time point, a 20-μl aliquot was removed and quenched with 10 μl 0.3 M HCl, followed by the addition of 10 μl of 450 mM Tris-HCl, pH 8.0. The amount of glutamate in the mixture was measured using the Glutamate-Glo assay (Promega; catalog no. J7022) according to the manufacturer’s instructions.

### Labeling and aminoacylation of ^32^P-labeled M. tuberculosis tRNA^Asn^.

The experiment was performed as previously described (42) with some modifications. M. tuberculosis tRNA^Asn^ (final concentration, 2.5 μM) was radiolabeled at 37°C for 30 min with 1 μM E. coli tRNA nucleotidyltransferase and [α-^32^P]ATP (final activity, 2 μCi/μl) (PerkinElmer; catalog no. BLU003X250UC) in 50 mM Tris-HCl, pH 8.0, 20 mM MgCl_2_, 5 mM dithiothreitol (DTT), and 50 μM sodium pyrophosphate (NaPPi). After phenol-chloroform (pH < 5.0) extraction, free [α-^32^P]ATP was removed from the sample via a MicroSpin G-25 column (Cytiva; catalog no. 27-5325-01), and then the tRNA was isopropanol precipitated at −20°C for 2 h. ^32^P-labeled tRNA^Asn^ (0.4 μM) was diluted with 12.5 μM unlabeled tRNA^Asn^, and this diluted mixture was used for aminoacylation assays as described above using unlabeled aspartate. The ^32^P-labeled Asp-tRNA^Asn^ pellet was dissolved in ddH_2_O to 80 μM and stored in aliquots at −20°C until needed.

### ^32^P nuclease P1 GatCAB amidotransferase assay.

The experiment was performed as previously described (42) with some modifications. The assay was performed in 40 mM HEPES-KOH, pH 7.5, 25 mM KCl, 8 mM MgCl_2_, 4 mM ATP, 1 mM glutamine, 10 nM GatCAB, and ^32^P-labeled Asp-tRNA^Asn^ (various concentrations from 0.25 to 8.0 μM). The reaction mixture was kept on ice until the 2-min preequilibration step at 37°C and then initiated with ATP and glutamine. A 5-μl aliquot for each time point was removed and quenched with 5 μl nuclease P1 (Sigma; product no. N8630) in a 100-mM sodium citrate suspension (pH 4.7, 0.66 mg/ml). The digestion was kept in a 37°C heat block for 30 min, and 3 μl of sample was carefully spotted onto prewashed polyethyleneimine (PEI)-cellulose thin-layer chromatographic (TLC) plates (Merck Millipore; catalog no. 105725). The TLC plate was eluted in developing buffer containing 10 mM NH_4_Cl and 5% acetic acid for 100 to 140 min. The air-dried plates were exposed to phosphor screens for at least 16 h and then quantified by Typhoon FLA 9500 phosphorimager (Cytiva).

### Gel filtration analysis.

Experiments were carried out at 4°C using an ÄKTA purifier and Superdex 200 Increase 10/300 GL column (Cytiva; catalog no. 28-9909-44). Five-hundred-microliter samples with indicated component concentrations were prepared in buffer G (50 mM HEPES-KOH, pH 7.2, 30 mM KCl, 6 mM MgCl_2_, and 1 mM DTT), incubated on ice for 30 min, and loaded onto the column equilibrated with buffer G. Peak fractions were pooled, concentrated using an Amicon Ultra-0.5 3-kDa centrifugal filter device (Merck Millipore; catalog no. UFC500324) at 4°C, and characterized by SDS-PAGE and native PAGE. For native PAGE analysis, an 8% polyacrylamide gel was prepared, run in buffer H (buffer G without 1 mM DTT) for 2 h at 90 V on ice, and stained with SYBR green II RNA gel stain (Solarbio; catalog no. SY1040). A Qubit protein assay kit was used to measure the protein concentration of the sample, which was then diluted to 0.5 mg/ml for the stability assays.

### *In vitro* stability assay.

The UNcle instrument (Unchained Labs) was used to measure the melting and aggregation of samples over a thermal ramp. We loaded 9 μl of 1.7 mg/ml GatCAB in buffer D or 0.5 mg/ml transamidosome in buffer G into a Uni (sample holder for 16 quartz capillary tubes used for analysis). Buffers were supplemented with 0.2/0.4 M trehalose as indicated. The temperature increased from 20 to 90°C in constant increments of 0.3°C/min, and the instrument generated both the BCM (barycentric mean of the intrinsic fluorescence spectra versus temperature) and aggregation curves (static light scattering intensity using lasers at 266 nm versus temperature). To determine Tm1, namely, the midpoint temperature of the first unfolding transition, the first-order derivative of the BCM curve was calculated and plotted against temperature to generate the derivative curve; the temperature corresponding to the first peak of the derivative curve represents Tm1. The aggregation onset temperature (Tagg_266_) was calculated by the UNcle Analysis software, with the Tagg_266_ peak threshold value set as 30%.

### Structural modeling of Mycobacterium tuberculosis Asn-transamidosome.

A model of the M. tuberculosis Asn-transamidosome was constructed based on the structure of the Pseudomonas aeruginosa transamidosome (PDB ID 4WJ3) (20). GatCAB and ND-AspRS sequences were from Mycobacterium tuberculosis H37Rv, and the structures were built using SWISS homology modeling (https://swissmodel.expasy.org). The tRNA^Asn^ sequence was from P. aeruginosa.

### *In vivo* mistranslation assay.

Mistranslation rates were measured in strains with M. tuberculosis *gatCA*-WT, G444S, and K61N constructed on an isogenic M. smegmatis *gatCA* deletion background with *Renilla*/firefly dual-luciferase reporters (9). The construct containing D120N in the *Renilla* luciferase sequence was used to measure the asparagine-to-aspartate mistranslation rate, while the construct containing K529R in the firefly luciferase sequence was used to measure the near-synonymous arginine-to-lysine mistranslation rate. The assay was carried out as previously described (7, 29, 31). Briefly, strains containing mistranslation reporters were grown to stationary phase (OD_600_ > 3) and diluted into fresh 7H9 medium supplemented with 0/0.5 mM trehalose to a final OD_600_ of ∼0.2. Expression of the reporter was induced with 100 ng/ml anhydrotetracycline (Clontech; catalog no. 631310) for 6 to 8 h. Bacteria were pelleted and lysed with passive lysis buffer provided in the dual-luciferase assay kit (Promega; catalog no. E1960), and luminescence was measured by a Fluoroskan Ascent FL luminometer (Thermo Scientific) according to the manufacturer’s instructions, with 1,000 ms as the integration time.

### Statistical analysis.

All experiments were performed at least three times independently. The number of repeats is shown in the figure legends. Data were analyzed with an unpaired, two-tailed Student's *t* test using GraphPad Prism. The results are shown as mean ± standard deviation. The level of significance was set to *P* < 0.05. *, *P* < 0.05; **, *P* < 0.01; ***, *P* < 0.001; ns, *P* > 0.05.
