# Supplementary material for: Clinically Relevant Mutations of Mycobacterial GatCAB Inform Regulation of Translational Fidelity
Source: mBio. 2021 Jul 6;12(4):e01100-21. doi: 10.1128/mBio.01100-21 (PMC8406222; doi:10.1128/mBio.01100-21)
Supplement: FIG S1 [file mbio.01100-21-sf001.pdf]

A

B

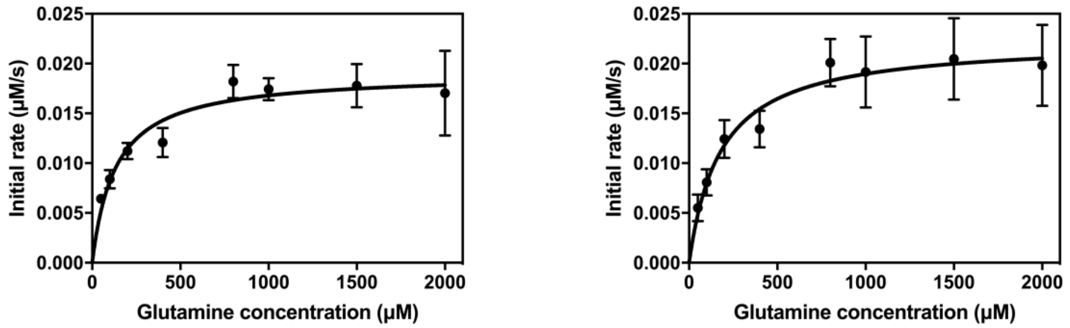

C

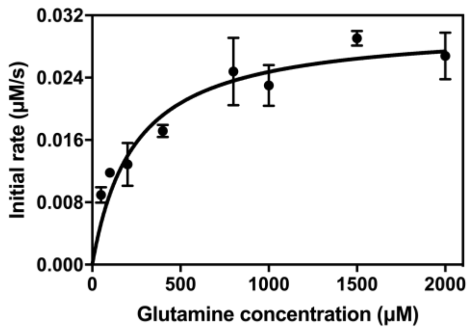

D

Glutamine concentration (μM)	*n* of different GatCAB groups		
	WT	G444S	K61N
50	4	5	3
100	4	5	3
200	4	5	7
400	5	6	5
800	4	4	5
1000	5	4	3
1500	4	4	3
2000	4	4	3

Supplementary Figure 1. Glutaminase assay of WT, G444S and K61N *M. tuberculosis* GatCAB. Michaelis-Menten curves of **(A)** WT **(B)** G444S and **(C)** K61N GatCAB glutaminase activity. Reactions were carried with 5 μM Asp-tRNA^{Asn}, containing ~20 nM GatCAB. GraphPad Prism was used to calculate kinetic parameters by nonlinear regression. Error bars represent the standard deviation of independent biological replicates, the *n* of which are listed in **(D)**.
