# Supplementary material for: Clinically Relevant Mutations of Mycobacterial GatCAB Inform Regulation of Translational Fidelity
Source: mBio. 2021 Jul 6;12(4):e01100-21. doi: 10.1128/mBio.01100-21 (PMC8406222; doi:10.1128/mBio.01100-21)
Supplement: FIG S2 [file mbio.01100-21-sf002.pdf]

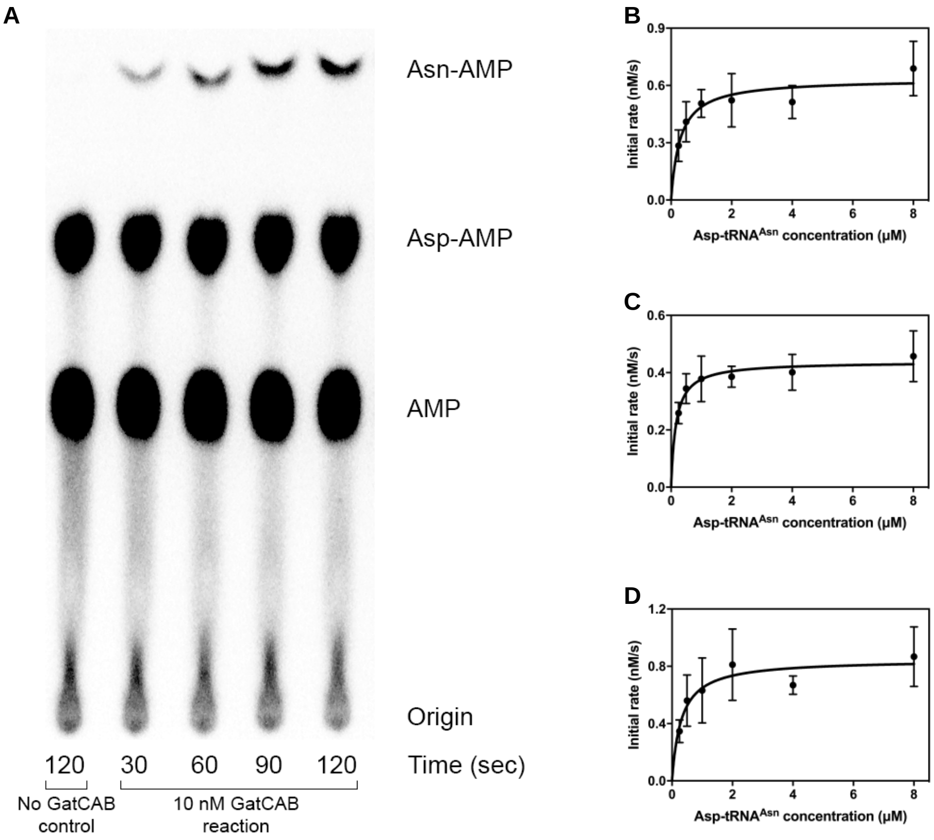


**Supplementary Figure 2. GatCAB amidotransferase assay with $^{32}$P-labeled tRNA.**
**(A)** Representative phosphorimage of the separation of Asn-[α-$^{32}$P]AMP, Asp-[α-$^{32}$P]AMP, [α-$^{32}$P]AMP, by PEI cellulose chromatography. Aliquots (5 µL) of the amidotransferase reaction at 37 °C (10 nM GatCAB from *M. tuberculosis* , 0.5 µM Asp-tRNA$^{Asn}$, 1 mM Gln, 4 mM ATP, 25 mM KCl, 8 mM $MgCl_2$, 40 mM HEPES-KOH pH 7.5) were taken at the time points indicated and quenched / digested at 37 °C with 5 µL of 100 mM sodium citrate pH 4.7, and 0.66 mg/mL of nuclease P1. Digested samples (3 µL) were then spotted onto a 20 × 20 cm PEI-cellulose glass plate, which was developed in 10 mM ammonium chloride, 5% acetic acid for ~2 hours. Aliquot from no GatCAB reaction at 120 seconds was taken as the background control. Michaelis-Menten curves of WT ($n$ = 4) **(B)**, G444S ($n$ = 4) **(C)** and K61N ($n$ = 3) **(D)** GatCAB are shown for their amidotransferase activity. GraphPad Prism was used to calculate kinetic parameters by nonlinear regression. Error bars represent the standard deviation of independent biological replicates.
