# Supplementary material for: Clinically Relevant Mutations of Mycobacterial GatCAB Inform Regulation of Translational Fidelity
Source: mBio. 2021 Jul 6;12(4):e01100-21. doi: 10.1128/mBio.01100-21 (PMC8406222; doi:10.1128/mBio.01100-21)
Supplement: FIG S3 [file mbio.01100-21-sf003.pdf]

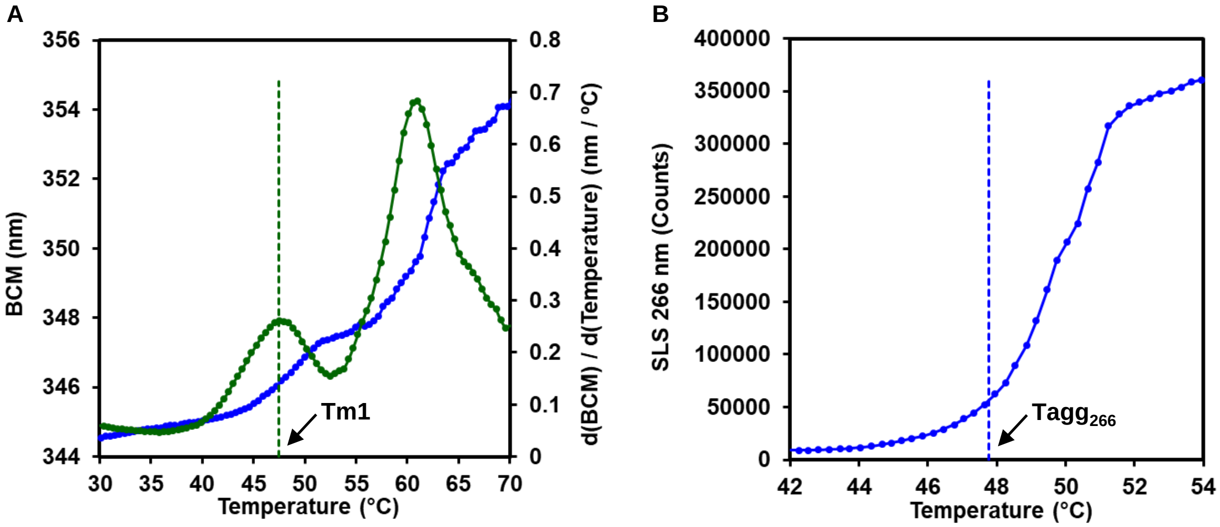

Supplementary Figure 3. Thermostability assay of *M. tuberculosis* GatCAB.

The assay was conducted on an UNcle instrument (Unchained Labs) by monitoring thermal melting and aggregation over a temperature range. **(A)** Representative Tm1 characterization of WT GatCAB using intrinsic fluorescence. At each temperature, the barycentric mean (BCM) of the intrinsic fluorescence spectra was measured and plotted against temperature to generate the BCM curve (blue). First-order derivative of the BCM curve was calculated and plotted against temperature (green). The temperature corresponding to the first peak of the first-order derivative curve was defined as Tm1, as shown by the dotted line (green). **(B)** Representative $Tagg_{266}$ characterization of WT GatCAB using static light scattering. At each temperature, the intensity of static light scattering at 266 nm (as represented by SLS 266 nm) was measured and plotted against temperature to generate an aggregation curve. Aggregation onset temperature ($Tagg_{266}$) of each protein was calculated by Uncle Analysis software, as shown by the dotted line.
