# Supplementary material for: Clinically Relevant Mutations of Mycobacterial GatCAB Inform Regulation of Translational Fidelity
Source: mBio. 2021 Jul 6;12(4):e01100-21. doi: 10.1128/mBio.01100-21 (PMC8406222; doi:10.1128/mBio.01100-21)
Supplement: FIG S4 [file mbio.01100-21-sf004.pdf]

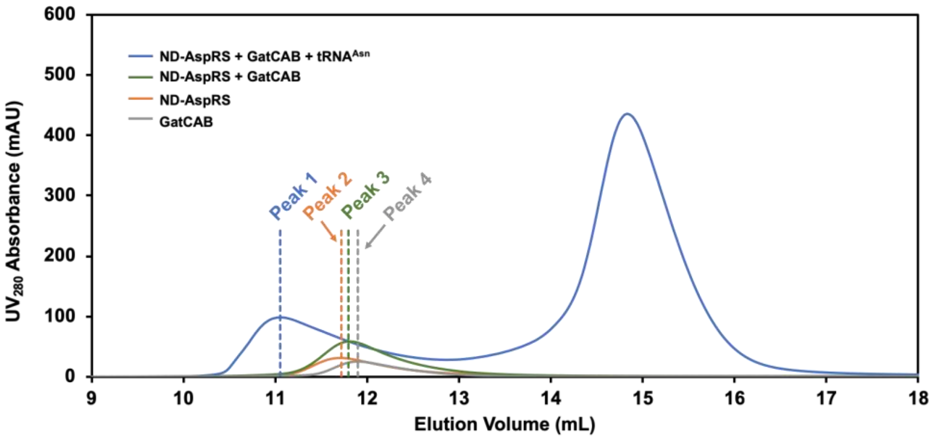

Supplementary Figure 4. Formation of the *M. tuberculosis* Asn-transamidosome is tRNA-dependent. Gel filtration conducted with free ND-AspRS, free GatCAB, a mixture of ND-AspRS and GatCAB, and a mixture of ND-AspRS, GatCAB and $tRNA^{Asn}$. 4 µM ND-AspRS, 2 µM WT GatCAB and 2 µM $tRNA^{Asn}$ were used.
