# Supplementary material for: Clinically Relevant Mutations of Mycobacterial GatCAB Inform Regulation of Translational Fidelity
Source: mBio. 2021 Jul 6;12(4):e01100-21. doi: 10.1128/mBio.01100-21 (PMC8406222; doi:10.1128/mBio.01100-21)
Supplement: TABLE S1 [file mbio.01100-21-st001.docx]

**SUPPLEMENTARY TABLES**

**Table S1. Bacterial strains used in this study.**

| Name | Descriptions | References or source |
| --- | --- | --- |
| *E. coli*, pET28a-AspS-CHis | Strain for expressing Mtb ND-AspRS, with C-terminal 6×His tag | This study |
| *E. coli*, pETDuet1-NStrep-GatCA-WT-GatB-CHis | Strain for expressing Mtb WT GatCAB, with N-terminal Strep tag II on GatC, and C-terminal 6×His tag on GatB | This study |
| *E. coli*, pETDuet1-NStrep-GatCA-G444S-GatB-CHis | Strain for expressing Mtb G444S GatCAB, with N-terminal Strep tag II on GatC, and C-terminal 6×His tag on GatB | This study |
| *E. coli*, pETDuet1-NStrep-GatCA-K61N-GatB-CHis | Strain for expressing Mtb K61N GatCAB, with N-terminal Strep tag II on GatC, and C-terminal 6×His tag on GatB | This study |
| *E. coli*, pTrc99a-T7-tRNA^Asn^ | Strain for transcripting Mtb tRNA^Asn^ under T7 promoter | This study |
| *M. smegmatis* KO::*gatCA*-WT, pTet-Ren-FF | Reporter strain where the WT *gatCA* gene is deleted and replaced elsewhere (L5 phage integration site) on the chromosome by Mtb WT *gatCA* gene, transformed with Renilla-Firefly dual luciferase construct | (9) |
| *M. smegmatis* KO::*gatCA*-WT, pTet-Ren-D120N-FF | Reporter strain where the WT *gatCA* gene is deleted and replaced elsewhere (L5 phage integration site) on the chromosome by Mtb WT *gatCA* gene, transformed with mutated Renilla-Firefly dual luciferase construct: measures Asn-to-Asp mistranslation | (9) |
| *M. smegmatis* KO::*gatCA*-WT, pTet-Ren-FF-K529R | Reporter strain where the WT *gatCA* gene is deleted and replaced elsewhere (L5 phage integration site) on the chromosome by Mtb WT *gatCA* gene, transformed with mutated Renilla-Firefly dual luciferase construct: measures Arg-to-Lys mistranslation | (9, 26) |
| *M. smegmatis* KO::*gatCA*-G444S, pTet-Ren-FF | Reporter strain where the WT *gatCA* gene is deleted and replaced elsewhere (L5 phage integration site) on the chromosome by Mtb G444S *gatCA* gene, transformed with Renilla-Firefly dual luciferase construct | (9) |
| *M. smegmatis* KO::*gatCA*-G444S, pTet-Ren-D120N-FF | Reporter strain where the WT *gatCA* gene is deleted and replaced elsewhere (L5 phage integration site) on the chromosome by Mtb G444S *gatCA* gene, transformed with mutated Renilla-Firefly dual luciferase construct: measures Asn-to-Asp mistranslation | (9) |
| *M. smegmatis* KO::*gatCA*-G444S, pTet-Ren-FF-K529R | Reporter strain where the WT *gatCA* gene is deleted and replaced elsewhere (L5 phage integration site) on the chromosome by Mtb G444S *gatCA* gene, transformed with mutated Renilla-Firefly dual luciferase construct: measures Arg-to-Lys mistranslation | (9, 26) |
| *M. smegmatis* KO::*gatCA*-K61N, pTet-Ren-FF | Reporter strain where the WT *gatCA* gene is deleted and replaced elsewhere (L5 phage integration site) on the chromosome by Mtb K61N *gatCA* gene, transformed with Renilla-Firefly dual luciferase construct | (9) |
| *M. smegmatis* KO::*gatCA*-K61N, pTet-Ren-D120N-FF | Reporter strain where the WT *gatCA* gene is deleted and replaced elsewhere (L5 phage integration site) on the chromosome by Mtb K61N *gatCA* gene, transformed with mutated Renilla-Firefly dual luciferase construct: measures Asn-to-Asp mistranslation | (9) |
| *M. smegmatis* KO::*gatCA*-K61N, pTet-Ren-FF-K529R | Reporter strain where the WT *gatCA* gene is deleted and replaced elsewhere (L5 phage integration site) on the chromosome by Mtb K61N *gatCA* gene, transformed with mutated Renilla-Firefly dual luciferase construct: measures Arg-to-Lys mistranslation | (9, 26) |
