# Supplementary material for: Clinically Relevant Mutations of Mycobacterial GatCAB Inform Regulation of Translational Fidelity
Source: mBio. 2021 Jul 6;12(4):e01100-21. doi: 10.1128/mBio.01100-21 (PMC8406222; doi:10.1128/mBio.01100-21)
Supplement: TABLE S2 [file mbio.01100-21-st002.docx]

**SUPPLEMENTARY TABLES**

**Table S2. Primers used in this study.**

| Name | Sequence (5’ to 3’) | Description |
| --- | --- | --- |
| *aspS*-F | TCTAGAAAGGAGATATACC**ATG**TTTGTGCTGCGCAGCCACG (*Xba*I underlined) | Used to amplify Mtb *aspS*-CHis (Rv2572c, encoding Mtb ND-AspRS, start codon changed to ATG) |
| *aspS*-R | AAGCTTTCATCAGTGGTGGTGGTGGTGGTGTGCCTGCTGGACCCGCTTG (*Hind*III and His_6_ tag underlined respectively) |  |
| NStrep-*gatCA*-F1 | GAAGGAGATATACC**ATG**TGGAGCCACCCGCAGTTCGAAAAGTCCCAGATCTCCCGCGAC (Strep tag II underlined) | First-step PCR to amplify Mtb NStrep-*gatCA* (start codon of *gatC* changed to ATG) |
| NStrep-*gatCA*-R1 | TTCCCCTATAGTGAGTCGTATTAGGTACCGAATTCTCATCAAATGGCGCTCAGTAGCGGG |  |
| NStrep-*gatCA*-F2 | CTTTAAGAAGGAGATATACCATGTG | Second-step PCR to amplify Mtb NStrep-*gatCA* |
| NStrep-*gatCA*-R2 | TTCCCCTATAGTGAGTCGTATTAG |  |
| *gatB*-CHis-F1 | CATCTTAGTATATTAGTTAAGTATAAGAAGGAGATATACC**ATG**ACTGTTGCTGCCGGGGCAG | First-step PCR to amplify Mtb *gatB*-CHis |
| *gatB*-CHis-R1 | TCATCAATGATGATGATGATGATGACCCTGCCCGCAGGCCTC (His_6_ tag underlined) |  |
| *gatB*-CHis-F2 | GACTCACTATAGGGGAATTGTGAGCGGATAACAATTCCCCATCTTAGTATATTAG | Second-step PCR to amplify Mtb *gatB*-Chis |
| *gatB*-CHis-R2 | GCAGCAGCCTAGGTTAACTCGAGTCATCAATGATGATGATGATGATGACCCTG |  |
| *gatA*-ATG-F | GGGGGATGAACA**ATG**ACGGACATCATCCGAT | Changing start codon of WT *gatA* to ATG using site-directed mutagenesis (SDM) |
| *gatA*-ATG-R | ATCGGATGATGTCCGTCATTGTTCATCCCCC |  |
| *gatA*-G444S-F | CTGCCGCTGAACTTGGCC**AGC**CACTGCGGCATGTCTG | Construction of pETDuet1-NStrep-GatCA-G444S-GatB-CHis using SDM |
| *gatA*-G444S-R | CAGACATGCCGCAGTGGCTGGCCAAGTTCAGCGGCAG |  |
| *gatA*-K61N-F | GCGGCCGCCATCGAC**AAT**CAGGTGGCCGCTGGAGAACC | Construction of pETDuet1-NStrep-GatCA-K61N-GatB-CHis using SDM |
| *gatA*-K61N-R | GGTTCTCCAGCGGCCACCTGATTGTCGATGGCGGCCGC |  |
| T7-tRNA^Asn^-F | CGGAATTC*taatacgactcactata*TCCCCTGTAGCTCAATTGGCAGAGCGTTCGGCTGTTAACCGAAG (*EcoR*I underlined; T7 promoter sequence in italics) | Construstion of pTrc99a-T7-tRNA^Asn^ |
| T7-tRNA^Asn^-R | CGGGATCCTGGCTCCCCCGGGAGGACTCGAACCTCCAACCCTTCGGTTAACAGCCGAACGCTCTG (*BamH*I underlined) |  |
